# Strength in Diversity: Integrating Community in Primary Health Care to Advance Universal Health Coverage

**DOI:** 10.9745/GHSP-D-21-00125

**Published:** 2021-03-15

**Authors:** Charlotte E. Warren, Ben Bellows, Rachel Marcus, Jordan Downey, Sarah Kennedy, Nazo Kureshy

**Affiliations:** a Population Council, Washington, DC, USA.; b U.S. Agency for International Development, Washington, DC, USA.; c Last Mile Health, New York, NY.; d Social Solutions International, supporting U.S. Agency for International Development, Washington, DC, USA.

## Abstract

The supplement highlights a systems approach that recognizes the communities' roles and their interactions with other health system actors to accelerate outcomes and reflect the diversity of the community health ecosystem. Several cross-cutting priorities emerge from the articles, namely coverage, community health financing, policy change, institutionalization, resilience, accountability, community engagement, and whole-of-society efforts.

## THE INTEGRAL ROLE OF COMMUNITY HEALTH IN ACHIEVING UNIVERSAL HEALTH COVERAGE

Approximately half of the world's population do not have access to essential health services.^1^^–^^3^ Recognizing the potential for community health to address gaps in coverage, financial protection, and access to quality care, the Declaration of Astana in 2018 committed to strengthening the role of community health in primary health care (PHC) as a means to accelerate progress toward universal health coverage (UHC).^4^^,^^5^ Before the Declaration of Astana, the transition from Millennium Development Goals to the Sustainable Development Goals (SDGs) also helped to reposition communities as resources for health systems strengthening and sources of resilience for individuals and families. A growing emphasis on the roles of communities recognizes community engagement, including community health workers (CHWs), as a means of realizing the full potential of PHC and the broader health system.^6^^,^^7^ CHW-delivered services are an integral component of responsive, accessible, equitable, and high-quality PHC.^8^

Countries are at the heart of the movement to renew political commitment for reenvisioned health systems that are capable of achieving UHC. Countries must mobilize the whole society—both public and private sectors as well as communities—as essential resources.^4^^,^^9^^,^^10^ There is a new urgency to design and operationalize the community component of PHC so that it can reach the most underserved, respond to pandemics, close the child survival gap, and accelerate the transformation of health systems in the next decade.

We underscore the significance of this special issue of *Global Health: Science and Practice* on community health, the first of its kind in the journal. The breadth of knowledge presented in this supplement exemplifies the progress made and persisting challenges in the global movement to redefine health systems and revitalize community-based PHC in the next decade. The 15 articles included in this supplement detail implementation experiences of designing, deploying, improving, scaling, and strengthening community health initiatives in PHC. The articles contain new methodologies, analysis, tools, and approaches that reinforce a systems-thinking lens in scaling and sustaining community health policies and programs in diverse contexts to achieve quality, equity, and efficiency. Learning from multiple countries and perspectives from across the globe, including findings on policy and practice and a new regional analysis from West and Central Africa and an update on financing trends in sub-Saharan Africa, highlight the challenges and opportunities in accelerating community-integrated PHC. Reflections on national progress from ministry of health representatives and key partners reinforce the urgent need to sustain political commitment, including addressing the financing gap and focusing on the “last mile.”

The knowledge acquired from these articles comes from implementing national priorities anchored in high-quality CHW platforms that were supported by collaborations such as the Integrating Community Health Collaboration (ICH) and the Community Health Roadmap.^10^^,^^11^ Several articles present the work of collaborators including ministries of health, nongovernmental partners, donors, and multilateral institutions in the ICH collaboration, which the U.S. Agency for International Development, United Nations Children's Fund, and the Bill and Melinda Gates Foundation supported in 7 countries (Bangladesh, Democratic Republic of the Congo, Haiti, Kenya, Liberia, Mali, Uganda). These articles provide an analysis of national directions that are needed to optimize policies and programs to engage communities and CHWs, and they demonstrate how local learning can strengthen linkages between communities and systems that improve quality, measurement, governance, and accountability in Bangladesh, Kenya, Liberia, and Mali.

To overcome barriers to achieving community health scale-up, more insight is needed into the functioning of community health programs (data and performance measures). Building on the ICH collaboration, a companion supplement in the *Journal of Global Health* (March 2021) focuses on the Community Health Worker Performance Measurement Framework developed by the Population Council and includes cross-country analyses using newly developed scales on trust in CHWs, CHW motivation, and related performance metrics.^12^ The work presented in both supplements advances national dialogue and decision making around key recommendations and complements the World Health Organization's Guideline on Health Policy and System Support to Optimize CHW Programs^12^^,^^13^ and the evidence generated in Exemplars in Global Health countries,^14^ both of which approach UHC objectives with the understanding that community members and community-based providers are critically important health system actors.

By synthesizing cross-country learning and showcasing the directions being taken by countries that are at the forefront, both of these supplements reflect the growing momentum to renew and advance the role of communities as the cornerstone of health systems. The intent is to use this emerging knowledge to bring an equilibrium to health systems with a greater community focus in the next decade.

## THE UNIQUE OPPORTUNITY FOR COMMUNITIES TO ACCELERATE HEALTH OUTCOMES

This GHSP supplement highlights a systems approach that recognizes the unique roles of communities and their interaction with other health system actors to accelerate outcomes and reflect the diversity of the community health ecosystem. We highlight the cross-cutting priorities for integrating the community within PHC that emerge from the articles: coverage, community health financing, policy change, institutionalization, resilience, accountability, community engagement, and whole-of-society efforts.

### Coverage of Populations

Multiple articles describe the results of developing assessment tools that support reform of community health systems and improve functional population coverage. Simen-Kapeu et al. conducted a bottleneck analysis of processes to strengthen and expand community health systems and strategies in 22 West and Central African countries^15^; the analysis identified gaps in community health financing, lack of equipment and supplies, and limited community ownership. Morrow et al. used the CHW Coverage and Capacity (C3) Tool to identify the required number of CHWs and their time allocation in Rwanda and Zanzibar, describing the importance of optimizing CHW investments by understanding the context and existing opportunities to engage with decision makers and stakeholders.^16^ Chen et al. describe how the institutional health reform cycle can guide policy makers in identifying gaps and opportunities while coordinating input from multiple stakeholders to extend CHW program coverage.^17^ These studies provide additional evidence that the likelihood of achieving the SDGs increases as coverage of underserved populations accessing lifesaving interventions and commodities improves.

### Community Health Financing

Globally, it is recognized that improved population health outcomes and economic performance are interlinked. Gichaga et al. reinforce this thinking and argue that an effective way for countries to build resilient health systems is to invest more in community-based PHC.^18^ For example, in sub-Saharan Africa, CHW investment has the potential to produce an economic return of up to 10:1.^19^ However, funding sources are rapidly shifting from being donor-led to a greater reliance on the domestic tax base in many countries, especially those entering middle-income status.^20^ Consistent with that accelerating shift, financing is described as a critical bottleneck to achieving maturity and scale in several articles.^15^^,^^17^^,^^18^^,^^21^ Saint-Firmin et al. analyze the distribution of reported expenditure, efficiency, and geospatial mapping in Mali to inform decision makers in transitioning to a domestically funded CHW program.^22^ They demonstrate where efficiencies could be found and targeted geographically to reach underserved communities. In Kenya, we see a need for additional research into the pathways through which health financing interventions support or hinder the success of community health programs.^23^

### Policy Change

Several articles, including those already mentioned,^15^^–^^17^ focus on policy change and explore the drivers of policy change in community health which, like any system, are complex at every level from global to regional and national to local. Hussein et al. and Healey et al. discuss the CHW policy development process in Liberia and Kenya.^21^^,^^23^ Two articles discuss how learning from vertical CHW programs drove evolution into broader and more integrated community programs. Palazuelos et al. describe adopting a community health model in 3 countries, broadening from a focus on individuals with HIV, multidrug resistant-TB, and noncommunicable diseases to a whole-household approach.^24^ Napier et al. look at the successes of reducing malaria in Lao People's Democratic Republic and Honduras and the need for expanding the roles and responsibilities of CHWs as community needs shift.^25^ Downey et al. stress the importance of orienting to policies that track CHW knowledge as a performance metric and that promote quality service delivery in CHW programs.^26^ Another critical policy priority is reducing CHW attrition by improving motivation and providing sufficient support.^27^

### Institutionalization

Institutionalizing community health within the PHC systems is also a common cross-cutting theme in the supplement. The institutional health reform cycle can guide policy makers in identifying gaps and opportunities, coordinating input from multiple stakeholders, to institutionalize CHW programing.^17^ Several articles explore the institutionalization of CHWs in terms of supportive supervision, remuneration, recognition, and referrals. Although the priorities vary across settings (e.g., career concerns, service delivery, and support), formal linkages to the broader health system are consistently important. Substantive change is more likely to occur when these performance domains of community health are institutionalized. Hussein et al. describe how the institutionalization of the community health services in Kenya occurred as a result of the extensive consultative process in developing the community health policy as well as increasing the visibility of the community strategy as a cornerstone of UHC.^23^

### Resilience

Other articles describe efforts to strengthen resilience and sustainability of community health as an effective layer of services in the health system. Several articles mention resilience of community health systems and CHWs especially in their response to earlier epidemics such as Ebola and polio and some success in mitigating the current coronavirus disease (COVID-19) crisis. Gichaga et al. describe how experience of contact tracing and home-based care by CHWs built on previous epidemics, but the high population density of informal urban communities make sustaining any gains a challenge.^18^ Healey et al. discuss how CHWs successfully responded to the Ebola virus disease outbreak of 2014–2016 in Liberia, which directly led to providing a strong platform for addressing COVID-19.^21^

### Community Engagement

Community engagement is a critical component within PHC. Community health has sometimes been incorrectly viewed as a field dominated by lay health workers and volunteers who move in and out of their positions in a state of impermanence. However, articles in this issue present evidence from across both the public and private sectors that are critical links in the broader health system and discuss CHW performance as essential for quality improvement. In particular, integrating community voices within monitoring and evaluation processes helps to triangulate with other data and galvanize policy makers to act. In some examples, processes were also developed to ensure information flows back to communities, improving both accountability and responsiveness.^21^^,^^28^ In Bangladesh, a community-clinic-centered-health service model helped to harmonize the work of different CHWs and improve accountability of both CHWs and community clinics to community members, which resulted in increased maternal health consultations and client satisfaction.^29^ In Mozambique, Amosse et al. discuss community engagement in setting up transport funds for obstetric and other emergencies.^30^ Community engagement, strengthening CHW capacity, and creating linkages with local transport infrastructure is critical to support access to the wider health system. Three articles describe specific challenges and opportunities on CHWs engaging communities around malaria, childhood malnutrition, and polio from diverse countries (Lao People's Democratic Republic, Honduras, Tajikistan, and India).^25^^,^^27^^,^^31^

### Whole-of-Society Efforts/Linkages

We recognize the roles of communities and their interaction with other health system actors that have the potential to accelerate outcomes and reflect the diversity of the community health ecosystem. Healey et al. describe Liberia's community health program's journey to scale after the Ebola virus disease outbreak in West Africa, highlighting the critical role of policy entrepreneurs and the importance of capitalizing on windows of opportunity to build strong coalitions.^21^ Palazuelos et al. discuss a cross-country learning process and the experience of a near-facility CHW program building trust between communities and health care teams.^24^ Two articles describe experiences from Kenya and include an in-depth discussion on the policy process over the last 15 years^23^ and the use of human-centered design to address a specific issue regarding the commodity supply chain among nomadic communities.^28^

## CONCLUSION

From the breadth of articles included in this supplement, it is clear that there is no single blueprint for strengthening a community health system but rather a range of processes, tools, and political commitment to support countries in their policy prioritization, financing, community engagement, and program rollout.

Community health is a significant component of PHC and driver of progress toward UHC, namely by expanding access to services, improving quality of interactions with the health system, and reducing out-of-pocket expenditure by promoting preventive health care. This year is important for reviewing national progress and country stock-taking. Translating the renewed political will and country roadmaps to strengthen the performance of a community-centered approach is fundamental to strengthening national systems that truly reach the most underserved, measure progress, increase equitable access to PHC, and ultimately make UHC a reality.
